# Expression and significance of CD44 and p-AKT in pancreatic head cancer

**DOI:** 10.1186/s12957-015-0746-8

**Published:** 2015-12-15

**Authors:** Li Xiaoping, Zhang Xiaowei, Zheng Leizhen, Guo Weijian

**Affiliations:** Department of Oncology, Xinhua Hospital, School of Medicine, Shanghai Jiaotong University, Shanghai, 200092 China; Department of Oncology, Cancer Hospital, Fudan University, Shanghai, 200032 China

**Keywords:** CD44, p-AKT, Pancreatic head cancer, Metastasis, Prognosis

## Abstract

**Background:**

CD44 and phosphorylated AKT (p-AKT) is a potentially interesting prognostic marker and therapeutic target in pancreatic cancer. The expression of CD44 and p-AKT has been reported to correlate with poor prognosis of pancreatic cancer in most literatures. The purpose of this study is to investigate the roles of CD44 and p-AKT in pancreatic head cancer and their correlation with the prognosis of pancreatic head cancer patients.

**Methods:**

Forty-eight pancreatic head cancer samples were collected dating from Jan. 2010 to Dec. 2012. Immunohistochemistry was applied to test the expression of CD44 and p-AKT in pancreatic head cancer. The clinical data of the patients were collected including their gender, age, the histology and location, lymph node metastasis, and so on. The correlation between the CD44 expression and the clinicopathological factors of patients with pancreatic head cancer was analyzed by the software SPSS 13.0.

**Results:**

The positive rates of CD44 and p-AKT expression in the samples were 64.6 and 29.2 %, respectively. There was a significant difference between the CD44 expression and the pancreatic cancer’ T staging, tumor node metastasis (TNM) staging, lymph node metastasis (*P* < 0.05). The Cox proportional hazard model showed that CD44 and lymph node metastasis were independent prognostic factors.

**Conclusions:**

CD44 was related to the distant metastasis and aggressive malignant behaviors of pancreatic head cancer.

## Background

Pancreatic cancer is a digestive tract malignant tumor with poor prognosis because of difficult diagnosis and rapid development, most of them located in the head of pancreas [1]. Most pancreatic head cancer patients have peripancreatic nerve invasion and lymph node metastasis during diagnosis, and 1-year survival rate is less than 20 % [2]. It is necessary to explore the biological index for the early diagnosis and prediction of pancreatic head cancer which present as aggressive and recurrent malignancies.

Tumor invasion and metastasis involves the interaction of multiple factors. Cancer stem cells (CSC), a subpopulation of tumor cells, are responsible for tumor initiation, growth, metastasis, and resistance to chemotherapy [3]. Some cell surface markers have been reported as CSC markers in pancreatic cancers, such as CD44, CD133, ALDH1, and ABCG2, and high expression of these markers is usually considered an indicator of poor prognosis [4]. CD44, an important marker for CSC, is a membrane glycoprotein involved in cell–cell and cell–extracellular matrix adhesion as well as cell migration, differentiation, and survival. CD44 may be involved in invasion and metastasis by regulating different signal transduction pathways, including phosphorylated AKT (p-AKT) [5]. p-AKT phosphorylates multiple proteins implicated in cellular processes leading to induction of cell survival and inhibition of apoptosis. This effect could be mediated by CD44. Previous studies demonstrated that anti-CD44 mAb induces apoptosis by suppressing the PI3K/Akt cell survival pathway [6].

Most scholars believe that the CD44 gene can be used as a new tumor marker, which greatly facilitates the early diagnosis of malignant tumor recurrence and metastasis [7, 8]**.** Several studies have reported overexpression of CD44 in subsets of pancreatic adenocarcinomas in 37–80 % of the tumors investigated [9]. However, most of them focused on all sites of the pancreas. There is relatively few data on CD44 expression in pancreatic head adenocarcinomas.

In this study,CD44 and p-AKT were selected as markers. Here, we used immunohistochemical (IHC) method to detect the expression of CD44 and p-AKT in the pancreatic head cancer tissues. The aim of the present study was to examine the prognostic relevance of CD44 expression in pancreatic head adenocarcinomas.

## Methods

### Patients and specimens

Forty-eight pancreatic head cancer samples were collected dating from Jan. 2010 to Dec. 2012. The clinical data of the patients were collected including their gender, age, the histology and location, lymph node metastasis, and so on. Standard demographic, clinicopathological, and tumor-specific data were collected retrospectively from hospital records, and the disease stages of the patients were classified according to the 2010 AJCC pancreatic cancer tumor node metastasis (TNM) staging system [10]. For the use of these clinical materials for research purposes, informed consent and approval from the Institute Research Ethics Committee was obtained.

### Immunohistochemistry and assessment

Four-micrometer sections of tissue were transferred to an adhesive-coated slide, and our pathologists have reviewed the slides to ensure that the tissues were consistent with pancreatic ductal adenocarcinoma (PDAC). Immunohistochemical staining to detect the expression of CD44 and p-AKT in paraffin sections was performed as described [11]. The quality of staining was judged in the control material from different organs, on the basis of the data available in the literature regarding gene/protein expression of CD44 and p-AKT in various tissue types. The number of positively stained cells and the intensity of positive staining were scored by two pathologists independently. Cases with different scores were discussed to reach an agreement. The intensity of staining was evaluated semiquantitatively as negative (no staining or staining in less than 10 % of cancer cells), lowly positive (11–25 %), or strongly positive (>25 %). Cells were considered positive only if CD44 and p-AKT intensity was lowly or strongly. Otherwise, the sample was considered negative. The immunostaining of each tissue was assessed in five areas of the acquired images of each tissue section, and the mean of these five scores was calculated. The whole sections were also screened at ×400 and ×1000 magnification, looking for features such as nuclear/cytoplasmic staining, expression in vessels, etc. The correlation between the expression of CD44, p-AKT, and the clinicopathological factors of patients with pancreatic cancer was analyzed.

### Statistical analysis

All statistical analyses were done by using the SPSS 13.0 software package (SPSS; SPSS Inc., Chicago, USA). In the set of IHC assay of paraffin-embedded tissue samples, the nonparametric test was used to estimate the correlations between CD44, p-AKT, and clinicopathologic characteristics. The Kaplan–Meier analysis module was used for comparing survival rates between multiple groups, and differences were measured using the log-rank test. Multivariate analysis of prognostic factors was performed using the Cox proportional hazard method. The results are presented as the median survival in months with 95 % confidence interval (CI), the relative risk with 95 % CI, and the number of patients at risk. A *P* value less than 0.05 was considered to be statistically significant as indicated.

## Results

### Clinicopathologic characteristics and outcomes

**Fig. 1:**
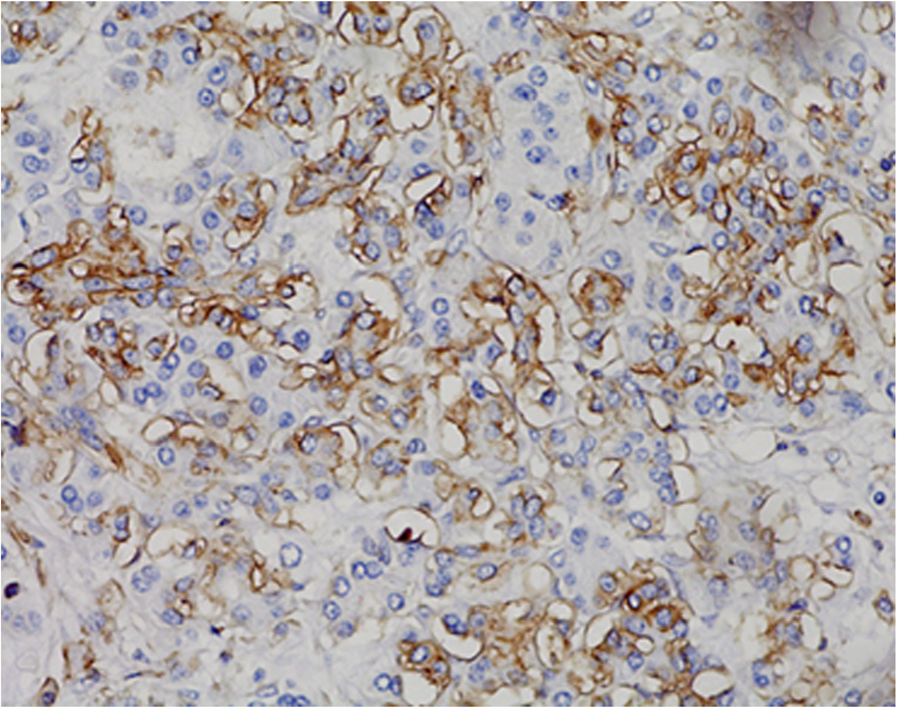
The expression of CD44 in pancreatic head cancer specimens

**Fig. 2:**
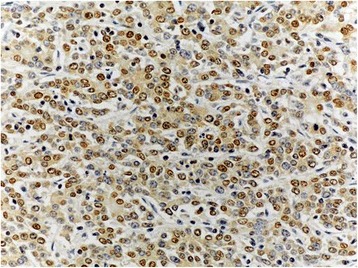
The expression of p-AKT in pancreatic head cancer specimens

By IHC analysis,31 (64.6 %) and 14 (29.2 %) paraffin-embedded archival pancreatic tumor tissues showed a positive staining for CD44 and p-AKT (Figs. 1 and 2). Patients’ clinical data between the CD44-positive and CD44-negative groups are listed in Table 1. Differences in age, sex, differentiation, vascular invasion, and nerve invasion between the two groups were not significant. Most patients had stage II disease (50 %); 35.4 % of patients had lymph node metastases. The majority of tumors were poorly differentiated (75.0 %), and the remaining tumors were well differentiated (10.4 %) and moderately differentiated (14.6 %). Forty-three patients received radical surgery. None of the patients received preoperative chemotherapy or chemoradiotherapy. Thirty followed by gemcitabine based postoperative adjuvant chemotherapy for patients with advanced stage (T3/4 or N1-3). Five patients were found to have liver or peritoneal metastases during operation and received palliative operation, followed by gemcitabine-based palliative chemotherapy.

**Table 1:** Relationship between expression of CD44, p-AKT, and clinicopathological parameters

Variable	Number	CD44		*P*	p-AKT		*P*
		Positive	Negative		Positive	Negative	
LNM				0.011			
Negative	31	15	16		6	25	0.056
Positive	17	15	2		8	9	
T classification				0.035			
T1-2	18	8	10		6	12	0.632
T3-4	30	23	7		8	22	
TNM stage							
I + II	28	13	15	0.002	14	14	0.613
III + IV	20	18	2		10	10	
Differentiation							
Well + moderately	12	9	3	0.492	3	9	0.516
Poorly	36	22	14		11	25	
Vascular invasion							
Negative	45	28	17	0.303	13	32	0.904
Positive	3	3	0		1	2	
Nerve invasion							
Negative	42	27	15	0.910	11	31	0.367
Positive	6	4	2		3	3	
Age (years)							
≥60	17	10	7	0.752	5	12	0.978
<60	31	21	10		9	22	
Sex							
Male	30	20	10	0.762	9	21	0.871
Female	18	11	7		5	13	

*LNM* lymph node metastasis

The nonparametric test was used for the relationship between the expression of CD44, p-AKT, and clinical-pathological factors. CD44-positive tumors were more likely associated with T stage (*P* = 0.035), TNM staging (*P* = 0.002), and lymph node metastasis (*P* = 0.011),which suggested that overexpression of CD44 correlated with a more aggressive phenotype in pancreatic head cancer. In pancreatic head cancer, p-AKT-positive cells were identified in most cases with various intensities in the positive cell population. However, to compare with CD44 expression patterns, we did not find any significant correlations with p-AKT expression.

### Univariate analysis of prognostic factors of pancreatic head cancer

**Fig. 3:**
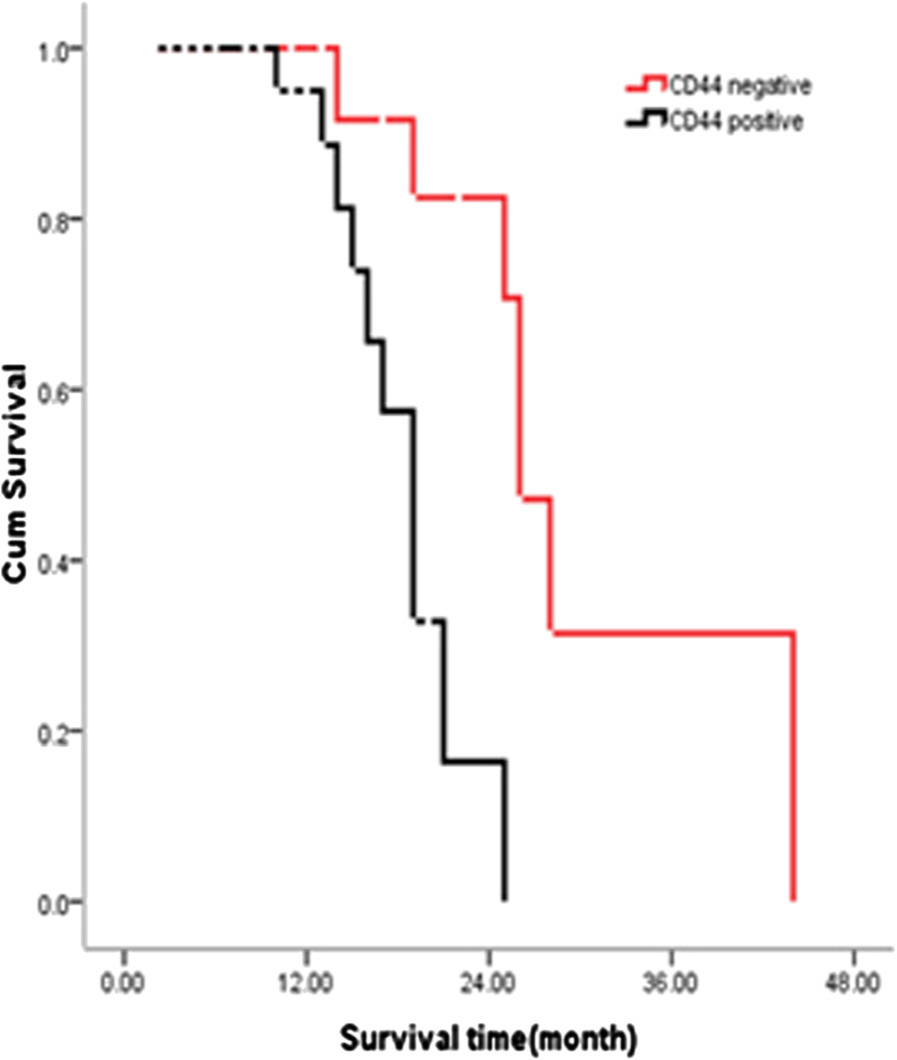
Effects of CD44 expression on pancreatic head cancer survival

All the patients were followed up to get the survival data. Overall survival time was defined as the time from surgery until death (living patients were censored at the time of their last follow-up). The median follow-up time was 39 months, and thirty patients had died at the last follow-up time. We examined the correlation of CD44 expression with patients’ survival of 48 pancreatic head cancers that had survival data available by Kaplan–Meier survival analysis (see Table 2). The median overall survival time of patients in the CD44-negative group were 30 months, whereas that in the CD44-positive group was only 18 months, and the difference between the two groups was significant (hazard ratio = 0.284; 95 % confidence interval, 0.125–0.407; *P* = 0.001). Log-rank test showed that T staging, lymph node metastasis, and neural invasion also significantly affect the prognosis of pancreatic head cancers. Advanced T staging, lymph node metastasis, and neural invasion positive patients survived shorter, and the difference is significant (*P* < 0.05). The survival difference between TNM staging, differentiation, vascular invasion, age, and sex were not statistically significant. The survival curve of the effects of CD44 expression on pancreatic head cancer is shown in Fig. 3. The results suggest that overexpression of CD44 correlates with poor prognosis in pancreatic head cancer.

**Table 2:** Univariate analysis of prognostic factors of pancreatic head cancer

	Hazard ratio	95 % CI	*P* value
CD44 (positive/negative)	0.284	0.125–0.407	0.001
p-AKT (positive/negative)	1.094	0.696–1.719	0.696
Lymph node metastasis (yes/no)	0.468	0.290–0.756	0.013
T classification (T1-2/T3-4)	0.446	0.283–0.702	0.000
TNM stage (I + II/III + IV)	1.216	0.772–1.916	0.393
Differentiation (well + moderate/poor)	1.019	0.650–1.599	0.984
Vascular invasion (yes/no)	1.513	0.965–2.371	0.087
Nerve invasion (yes/no)	0.408	0.246–0.676	0.000
Age (<60/≥60)	1.454	0.930–2.282	0.873
Sex (male/female)	1.255	0.798–1.974	0.345

**Fig. 4:**
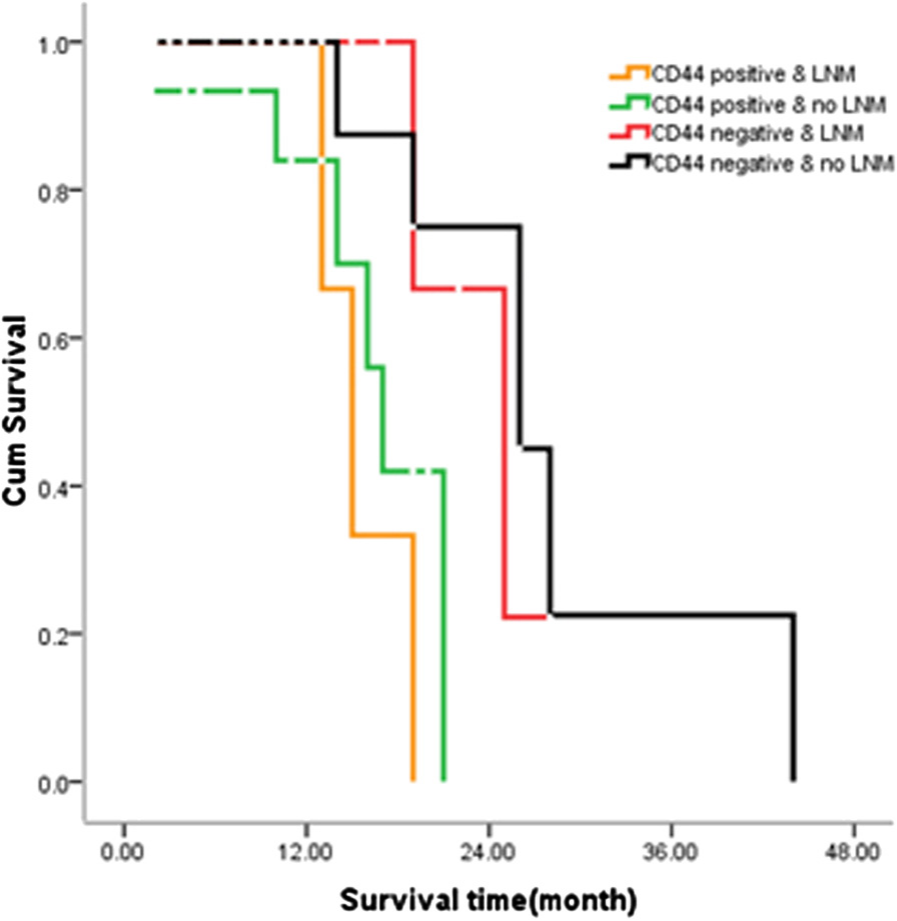
Effects of CD44 expression and lymph node metastasis on pancreatic head cancer survival

Since tumors expressing CD44 were significantly more likely to be lymph node metastasis than CD44-negative tumors, the joint effects of CD44 status and lymph node metastasis on survival were assessed by Kaplan–Meier analysis, stratifying for CD44 status (positive vs*.* negative) and lymph node metastasis (positive vs*.* negative) (Fig. 4). Patients whose tumors overexpressed CD44 and lymph node metastasis (LMN) had significantly poorer survival than CD44 and LMN groups (*P* = 0.001).

### Multivariate analysis of prognostic factors of pancreatic head cancer

In a multivariable Cox proportional hazard model, which included lymph node metastasis, clinical stage, CD44 expression, and nerve invasion, CD44-positive tumors and lymph node metastasis independently predicted poor prognosis. Patients with CD44-positive expression had worse overall survival compared with patients with CD44-negative expression (hazard ratio = 0.199; 95 % confidence interval, 0.049–0.965; *P* = 0.045) (Table 3). There was a strong correlation between CD44 expression and lymph node metastasis (*P* = 0.002). These data suggest that the CD44 and lymph node metastasis were the independent prognostic indicator.

**Table 3:** Multivariate analysis of prognostic factors of pancreatic head cancer

	Hazard ratio	95 % CI	*P* value
CD44 (positive/negative)	0.199	0.049–0.965	0.045
T classification (T1-2/T3-4)	0.125	0.117–0.891	0.059
Lymph node metastasis (yes/no)	0.299	0.015–1.552	0.023
Nerve invasion (yes/no)	0.119	0.043–0.327	0.933

## Discussion

Because of its poor prognosis, pancreatic cancer is one of the four or five most common causes of cancer mortality in developed countries [12]. Lymph node metastasis is a poor prognostic factor in patients with pancreatic cancer. The negative effect of lymph node metastasis as a prognostic factor for patients undergoing surgical resection for pancreatic head adenocarcinoma has been well established [13]. Currently, the only biomarker used in the routine management of pancreatic head cancer is CA19-9. But approximately 5 % of the population do not secrete CA19-9 [14]. Therefore, much effort has focused on enhancing the performance of CA19-9 by including it within larger panels of markers.

CD44 has been studied for three decades, but no consensus opinion on cancer progression has been reached until now. CD44 is the major hyaluronan receptor. Invasive and metastatic growth can be mediated through the interaction of cell surface CD44 with hyaluronan or cell–cell interactions [15]. CD44 was revealed to be a target of the Wnt pathway, which is accepted as a key pathway for the stemness maintenance of cancer stem cell markers [16]. Recent clinical studies have shown high levels of CD44 expression in gastric cancer, colorectal cancer, and nonsmall cell lung cancer [17–19]. It was reported that overexpression of CD44 indicated bad clinical features and poor prognosis. Moreover, a large body of epidemiological, clinical, and molecular evidence suggests that CD44 was overexpressed in pancreatic cancer cell lines and pancreatic tumors and plays an important role in the carcinogenesis and progression of pancreatic cancer [20, 21]. Cell surface expression of CD44 plays an important role in the defense against reactive oxygen species, leading to ultimate survival of CSCs. Jiang et al. [22] provide in vivo evidence that CD44 is required for pancreatic cancer invasion and CD44 regulates pancreatic cancer cell invasion through MT1-MMP. Li et al. [23] also reported that increased CD44v expression was found in metastatic pancreatic carcinoma in human tumor tissue. Clinical analysis showed that CD44v6+ and CD44v9+ were correlated with lymph node metastasis, liver metastasis, and TNM stage.

In the present study, we show that CD44 is overexpressed in pancreatic head cancer tissues. Importantly, we found that overexpression of CD44 correlated with advanced clinical stage and positive lymph node metastasis. Our previous study also showed that stable knockdown of CD44 expression in pancreatic cancer cells can inhibit proliferation and migration in pancreatic cancer cells. This provide preliminary direct evidence for the possibility of CD44 regulating the metastasis of pancreatic cancer. These data clearly suggest that CD44 not only plays a key role in tumorigenesis but may also be involved in the progression and metastasis of pancreatic head cancer.

Lymph node involvement in pancreatic head cancer is one of the strongest adverse prognostic factors, with 5-year survival rate falling significantly to less than 10 % in cases of metastatic lymph node. Many articles have proved that CD44 was closely related with lymph node metastasis [24], which was well supported by our report. The results of our report supported that the function of the lymph node metastasis might be dependent on CD44. The finding in the present study that CD44 expression correlates with a favorable prognosis in pancreatic cancer can be explained by the fact that there is a significant association between CD44 expression and lymph node metastasis. In human pancreatic cancer tissue, lymph node metastasis overexpressed CD44 while there was less expression of CD44 in negative lymph node metastasis. More than 80 % of tumors with lymph node metastasis showed overexpression of CD44. This result is consistent with previous studies. For more detailed analysis, we compared between the patients with CD44-positive and lymph node metastasis. We found that the subgroup of patients with CD44-negative/no lymph node metastasis tumors had a significantly better survival compared to patients with CD44-positive/lymph node metastasis tumors. Multivariate Cox proportional hazard model analysis showed the strong statistical association between CD44 expression and lymph node metastasis. Thus, the presence of CD44 expression in these tumors appears to be a marker of favorable prognosis closely linked to the lymph node metastasis.

CD44-positive cells constitute the resistant cell population, and CD44 could be a therapeutic target to overcome the drug resistance for pancreatic cancer. Using an antibody targeting CD44s in mice with human pancreatic tumor xenografts, Li et al. [25] found that anti-CD44s reduced tumor growth and metastasis. The antibody also reduced the number of tumor initiating cells in cultured pancreatic cancer cells and inhibited cell proliferation and survival signaling.

The relationship between the expression of p-AKT and tumor prognosis remains controversial [26, 27]. Liu et al. [28] showed that the positive expression rate of p-AKT in pancreatic cancer was 83.8 %. The related research in most of pancreatic cancer, high expression of p-AKT, is correlated with poor prognosis [29], but there are also some research considered p-AKT-positive staining is correlated with better prognosis. We did not find any significant correlations between high p-AKT expression and certain clinicopathological findings. The limitations could be due to the limited number of samples in our study. Further studies are needed to explore the mechanisms of p-AKT in pancreatic head cancer.

## Conclusions

In summary, overexpression of CD44 was associated with poor overall survival in patients with pancreatic head cancer. However, more prospective studies are needed to explore the prognostic value of CD44 in pancreatic head cancer. With the thoroughly research in the mechanism and regulation pathway, CD44 will play a greater role in tumor diagnosis, treatment, and prognosis.

## Abbreviations

CI: confidence interval
CSC: cancer stem cells
LMN: lymph node metastasis
PDAC: pancreatic ductal adenocarcinoma
